# Rational evaluation of computed tomography scans using a risk-adjusted model in a comprehensive public tertiary hospital in China

**DOI:** 10.3389/frhs.2026.1715517

**Published:** 2026-02-04

**Authors:** Jia Guo, Xiaoguang Yang, Yueming Zhang, Pengfei Zhou, Lin Shu, Haibo Liu, Kuanyu Xu, Hanbo Zhang, Huahe Zhang, Hong Jia, Yunming Li

**Affiliations:** 1 School of Public Health, Southwest Medical University, Luzhou, China; 2 Office of Medical Information and Data, Medical Support Center, The General Hospital of Western Theater Command, PLA, Chengdu, China; 3 East-District Out-Patient Section, State Key Laboratory of Oral Diseases, National Clinical Research Center for Oral Diseases, West China Hospital of Stomatology, Sichuan University, Chengdu, China; 4 Department of Health Economics, The General Hospital of Western Theater Command, PLA, Chengdu, China; 5 Department of Medical Engineering, Medical Support Center, The General Hospital of Western Theater Command, PLA, Chengdu, China; 6 Department of Information, Medical Support Center, The General Hospital of Western Theater Command, PLA, Chengdu, China; 7 Collaborating Center of the National Institute of Health Data Sciences, Southwest Medical University, Luzhou, China

**Keywords:** China, computed tomography, MDC, rational evaluation, risk-adjusted model

## Abstract

**Background:**

In the context of medical insurance payment reform in China, Computed tomography (CT), as a key type of large medical equipment, currently faces challenges of over-scanning or under-scanning. This study aims to identify the factors influencing the number of CT scans, perform risk adjustment on the number of CT scans, and evaluate the rationality of the number of CT scans for each major diagnostic category (MDC).

**Methods:**

In the public tertiary general hospital in Sichuan Province, the top 10 MDCs with the highest total number of CT scans in 2023 were selected. A risk-adjusted model was used to estimate the expected number of CT scans. The utilization of CT scans was classified as over-scanning, under-scanning, and rational scanning based on the ratio of observed to expected scan numbers.

**Results:**

The top 10 MDCs included 29,461 encounters and 37,672 CT scans. The number of CT scans varied across different MDCs: five exhibited over-scanning, three showed under-scanning, and two demonstrated rational scanning. The risk-adjusted model revealed that age, admission condition, first-time hospitalization, medical insurance, and length of stay were statistically significant in determining both the decision to perform a CT scan and the number of CT scans conducted.

**Conclusions:**

This study evaluated the rationality of the number of CT scans across the top 10 MDCs, established a methodological framework for hospital to explore the rationality of the number of other medical examinations.

## 1 Introduction

In June 2020, The National Healthcare Security Administration issued the “Notice on Printing and Distributing the Refined Grouping Scheme for Healthcare Security Diagnosis-Related Groups (CHS-DRG)” (1), marking a new stage in the reform of medical insurance payment. Medical examinations are an indispensable component of modern healthcare services (2). Large medical equipment constitutes a significant portion of medical examinations and is of high value, playing a crucial role in disease prevention, diagnosis, treatment, and patient rehabilitation. Internationally, efforts to control healthcare costs by reducing the use of unnecessary large-scale medical equipment have had limited success, as seen in the United States (3). Likewise, in Japan, the imaging referral guidelines provided by the Japanese Radiological Society have not substantially modified established physician practices or fully addressed the inappropriate use of major diagnostic equipment (4). Computed tomography (CT) is the most widely used and efficient imaging examination method in clinical practice, stands out among large medical equipment in terms of both application scale and cost proportion (5). Over the past two decades, China has witnessed a substantial increase in the number of medical institutions and diagnostic radiology devices per million population, with the number of CT scanners growing by nearly fivefold (6). While the widespread adoption of CT examinations has significantly improved diagnostic efficiency (7). However, the implementation of new payment models has exerted complex effects on medical practices, posing new challenges to the rational use of CT scans.

On the one hand, under the CHS-DRG system, to maintain the total cost cap, increase the weight of benchmark diagnosis groups, and elevate the case mix index (CMI), unnecessary or excessive tests or treatments are employed to inflate healthcare resource consumption (8). The centralized procurement policy (9) reforms in China have led to significant reductions in the prices of pharmaceuticals and medical consumables. However, the adjustments made to medical service prices—intended to reflect the value of healthcare professionals' technical services—have not met expectations. In response to revenue losses stemming from decreased drug and consumable prices, hospitals may resort to excessive examinations as a means of maintaining overall revenue (10). This over-scanning of CT scans not only exposes patients to unnecessary radiation exposure (11–13), but also lead to incidental findings and false-positive results that necessitate additional scans (14).

On the other hand, under the CHS-DRG system, medical insurance no longer reimburses healthcare institutions based on the actual hospitalization costs; instead, payments are determined according to established standards for each case within the CHS-DRG framework (15).When public hospitals are unable to balance CHS-DRG payment standards with the costs of diagnosis and treatment, their inpatient medical insurance reimbursements often fall short of actual expenses, resulting in financial losses that hospitals must absorb (8). Consequently, physicians may adopt more conservative practices during treatment, potentially leading to a reduction in the number of necessary CT scans (16).

Risk adjustment is a statistical method used to study the impact of various combined factors on outcome variation. It enables fair comparisons between healthcare institutions by accounting for confounding factors (17). Although risk-adjusted models have been widely applied internationally to evaluate healthcare quality (18) and surgical safety (19), their potential in analyzing the appropriateness of medical imaging remains underexplored. Current research has mainly focused on descriptive analyses of CT scan rates. For instance, Lodwick et al. (20) analyzed CT scan utilization in tertiary pediatric hospitals using All Patient Refined Diagnosis Related Group (APR-DRG) and disease severity levels for risk adjustment. Their findings indicated that after risk adjustment using a generalized linear model, the CT scan rates of various hospitals varied, and the gap between the hospital with the lowest scan rate and the one with the highest scan rate narrowed after risk adjustment. Parker et al. (21) employed a generalized estimating equation model to adjust for risk by incorporating multiple covariates at the hospital level, such as the CMI, to obtain the adjusted CT scan rates and evaluate changes in CT scan rates among hospitalized children from 2004 to 2012. They concluded that CT scan rates had decreased, emphasizing the importance of risk adjustment in evaluating the rationality of medical device use. However, there is a lack of validation of the necessity of individualized scans after risk adjustment. Furthermore, there is a notable gap in the evaluation of CT scan rationality within single medical institutions, which is particularly critical for hospital administrators under the CHS-DRG payment system. Such analysis enables hospitals to understand the basic situation of hospital CT scans and evaluate their appropriateness.

This study aims to address the key issue of evaluating the rationality of CT scans under the CHS-DRG system. By applying a risk adjustment approach to control for confounding factors such as case complexity, we seek to (1) evaluate the rationality of the number of CT scans across different Major Diagnostic Categories (MDCs) and (2) establish a methodological framework for evaluating the rational use of other medical equipment in hospitals. Our method enables the identification of both overuse and underuse of medical equipment from a data perspective, providing policymakers and hospital administrators with scientifically validated tools.

## 2 Materials and methods

### 2.1 Study population

**Figure 1:**
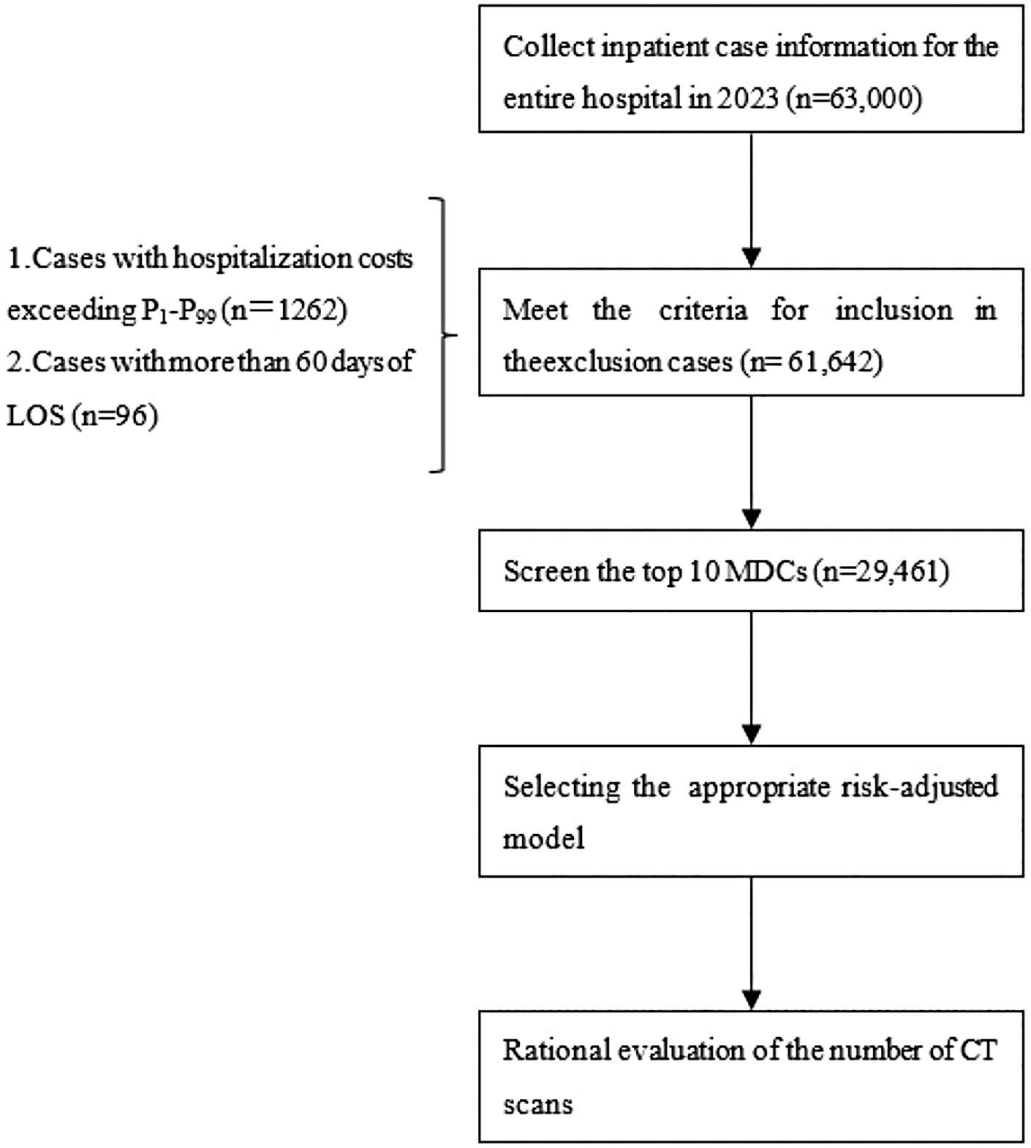
Flowchart for population selection and rationality evaluation of the number of CT scans.

We conducted a retrospective study of all inpatients with complete medical records in the information systems of a public tertiary general hospital in Sichuan Province from January 1 to December 31, 2023. To ensure data quality, the following exclusion criteria were applied: patients with total hospitalization costs outside the 1st to 99th percentile range of the overall distribution, and patients with a length of stay (LOS) exceeding 60 days (22), because CHS-DRG is not applicable to long-term hospitalization cases (22). All data were anonymized to protect information security and patient privacy. Based on the CHS-DRG framework, we selected the 10 MDCs with the highest number of CT scans in 2023 for analysis (Figure 1).

### 2.2 Definition of the number of CT scans

According to the regulations specified in the Sichuan Province Medical Service Price Project Compilation Standards, each anatomical region is counted as one scan. Bilateral examinations of paired anatomical structures are recorded as two separate scans. CT scans are categorized into the following anatomical regions: head, neck, chest, entire abdomen, pelvis, spine, extremities, and regions others.

### 2.3 Determination of risk adjustment factors

Based on a comprehensive literature review (20–25), clinical expertise, and research objectives, the following variables were selected: gender, age, admission condition (dangerous, urgent, stable). The admission condition is defined as follows: the clinical condition of a patient upon admission prior to undergoing any imaging examinations. Patients marked as “dangerous” are classified as critically ill patients. Patients with an admission status marked as “urgent” are classified as emergency patients. Patients with an admission status marked as “stable” are classified as routine patients. First-time hospitalization (no, yes), critical and serious condition (no, yes), medical insurance (no, yes), complication or comorbidity (none, mild, severe) This indicator grades the severity of other diagnoses in a case based on the list of comorbidities or complications in the National CHS-DRG Subgroup Classification. First, the case's other diagnoses are matched against the list of severe comorbidities or complications. If a match is found, the case is defined as having “severe comorbidities or complications.” If no severe comorbidities or complications are matched, the case is then matched against the list of mild comorbidities or complications. A successful match defines the case as having “mild comorbidities or complications present.” If no matches are found in either list, the case is defined as having “no comorbidities or complications.” (1). Admission method (outpatient, emergency), LOS, surgery (including other procedures), and CMI. The calculation of the CMI value involved extracting the DRG weights for all discharged cases at the hospital in 2023. These weights were automatically computed by the official DRG grouper based on patient information, including primary diagnosis, secondary diagnoses, surgical procedures, age, and gender (1).

### 2.4 Methods for selecting risk adjustment models

Four count models—Poisson regression, negative binomial regression, zero-inflated Poisson regression, and zero-inflated negative binomial regression—were selected as risk adjustment models based on distribution characteristics of the number of CT Scans.

Statistical analysis employed the O test and Vuong test to evaluate the distribution characteristics of the number of CT scans. The O test was used to detect overdispersion in the number of CT scans. When O equal to or was above than 1.96, it indicated that the number of CT scans was over-dispersed (26). A Vuong test statistic (Z value) equal to or greater than 1.96 suggested the presence of excessive zero counts in the number of CT scans (27). Then compared the goodness of fit of the four models and used the above method to determine which model to choose as the risk-adjusted model.

### 2.5 Evaluation of the rationality of the number of CT scans

For each MDC, the observed-to-expected (O/E) ratio for the number of CT scans was calculated. The observed number of CT scans for each MDC served as the numerator, while the expected number—derived from a risk-adjusted model—served as the denominator.

The O/E ratio and its 95% confidence interval (CI) were calculated for each MDC using the Wald method (28), and internal validation was performed via bootstrapping to ensure robustness of the results. To evaluate the rationality of the number of CT scans within each MDC, the following approach was adopted: If the 95% CI fully included the value 1, the observed number of CT scans was consistent with the expected number, indicating rational scanning. If the 95% CI was entirely above 1, the observed number was significantly higher than expected, suggesting potential over-scanning. If the 95% CI was entirely below 1, the observed number was significantly lower than expected, suggesting potential under-scanning (29).

### 2.6 Statistical analysis

For normally distributed quantitative data, mean ± standard deviation (*x̅* ± s) was used. For non-normally distributed quantitative data, median and interquartile range M (P_25_, P_75_) was used. Categorical variables were described using frequency or proportion. All statistical analyses were conducted using R software (version 4.4.2). A *P* value less than 0.05 is considered statistically significant.

## 3 Results

### 3.1 Description of the study population headings

Considering sample size and clinical representativeness, the following 10 Major Diagnostic Categories (MDCs) were ultimately selected: MDCA (pre-grouped diseases and related procedures), MDCB (neurological diseases and functional disorders), MDCF (cardiovascular diseases and functional disorders), MDCG (gastrointestinal diseases and functional disorders), MDCH (liver, biliary tract, and pancreas diseases and functional disorders), MDCI (musculoskeletal diseases and functional disorders), MDCK (endocrine, nutritional, and metabolic diseases and functional disorders), MDCS (infectious and parasitic diseases), MDCT (mental disorders and functional disorders), and MDCZ (multiple severe injuries).

The hospital recorded a total of 63,000 patient encounters and 56,615 CT scans in 2023. The mean number of CT scans per person was 0.90 ± 1.22. Among these, the top 10 MDCs accounted for 29,461 encounters, representing 46.76% of the total number of encounters in the hospital, and 37,672 CT scans, accounting for 66.54% of the hospital's total number of CT scans, with an average of 1.28 ± 1.42 scans per person.

Table 1 presents the demographic and clinical characteristics of the study sample across the top 10 MDCs (*n* = 29,461). Among them, 45.66% of the patients were female, and the mean age was 57.52 ± 15.85 years. 68.17% of patients were admitted in stable condition, and 30.04% of patients were first-time hospitalization. The majority (82.07%) of patients did not have critical or severe conditions. Nearly 90.81% of patients were covered by medical insurance. The median LOS was 7.01 (4.79, 11.13) days. The median number of surgical procedures was 1 (0, 3), and the median CMI was 0.90 (0.65, 1.89).

**Table 1:** Characteristics of the study sample (*n* = 29,461).

Characteristic	Total	MDCA	MDCB	MDCF	MDCG	MDCH	MDCI	MDCK	MDCS	MDCT	MDCZ
Gender, *n* (%)											
Female	13,452 (45.66)	104 (36.75)	1,934 (43.38)	2,651 (43.20)	2,358 (40.28)	3,088 (48.33)	2,065 (52.41)	933 (56.17)	182 (43.75)	93 (51.10)	48 (33.80)
Male	16,009 (54.34)	179 (63.25)	2,524 (56.62)	3,485 (56.80)	3,496 (59.72)	3,301 (51.67)	1,875 (47.59)	728 (43.83)	234 (56.25)	89 (48.90)	94 (66.20)
Age, years	57.52 ± 15.85	60.54 ± 19.32	59.24 ± 16.65	61.56 ± 14.81	58.39 ± 15.27	55.17 ± 14.67	53.94 ± 16.47	52.93 ± 14.76	55.62 ± 21.61	55.19 ± 18.84	54.68 ± 16.44
Admission condition, *n* (%)											
Dangerous	681 (2.31)	43 (15.19)	211 (4.73)	242 (3.94)	59 (1.01)	82 (1.28)	8 (0.20)	11 (0.66)	11 (2.64)	2 (1.10)	12 (8.45)
Urgent	8,696 (29.52)	143 (50.53)	2,014 (45.18)	1,842 (30.02)	1,260 (21.52)	1,879 (29.41)	901 (22.87)	281 (16.92)	215 (51.68)	54 (29.67)	107 (75.35)
Stable	20,084 (68.17)	97 (34.28)	2,233 (50.09)	4,052 (66.04)	4,535 (77.47)	4,428 (69.31)	3,031 (76.93)	1,369 (82.42)	190 (45.67)	126 (69.23)	23 (16.20)
First-time hospitalization, n(%)											
No	8,852 (30.04)	124 (43.82)	1,231 (27.61)	1,968 (32.07)	1,735 (29.64)	1,975 (30.91)	1,159 (29.42)	407 (24.50)	167 (40.14)	63 (34.63)	21 (14.79)
Yes	20,609 (69.96)	159 (56.18)	3,227 (72.39)	4,168 (67.93)	4,119 (70.36)	4,414 (69.09)	2,781 (70.58)	1,254 (75.50)	249 (59.86)	119 (65.38)	121 (85.21)
Critical and serious condition, *n* (%)											
No	24,180 (82.07)	28 (9.89)	3,248 (72.86)	4,429 (72.18)	5,293 (90.42)	5,299 (82.94)	3,808 (96.65)	1,517 (91.33)	274 (65.87)	165 (90.66)	119 (83.80)
Yes	5,281 (17.93)	255 (90.11)	1,210 (27.14)	1,707 (27.82)	561 (9.58)	1,090 (17.06)	132 (3.35)	144 (8.67)	142 (34.13)	17 (9.34)	23 (16.20)
Medical insurance, *n* (%)											
No	2,706 (9.19)	19 (6.71)	536 (12.02)	264 (4.30)	388 (6.63)	347 (5.43)	875 (22.21)	94 (5.66)	57 (13.70)	26 (14.29)	100 (70.40)
Yes	26,755 (90.81)	264 (93.29)	3,922 (87.98)	5,872 (95.70)	5,466 (93.37)	6,042 (94.57)	3,065 (77.79)	1,567 (94.34)	359 (86.30)	156 (85.71)	42 (29.58)
Complications or comorbidity, *n* (%)											
None	13,420 (45.55)	18 (6.36)	1,810 (40.60)	3,136 (51.11)	2,986 (51.01)	2,505 (39.21)	2,080 (52.79)	640 (38.53)	168 (40.38)	78 (42.86)	2 (1.41)
Mild	10,067 (34.17)	67 (23.67)	1,695 (38.03)	1,446 (23.57)	1,890 (32.29)	2,455 (38.43)	1,412 (35.84)	813 (48.95)	141 (33.89)	67 (35.81)	78 (54.93)
Severe	5,974 (20.38)	198 (69.96)	953 (21.38)	1,554 (25.33)	978 (16.71)	1,429 (22.37)	448 (11.37)	208 (12.52)	107 (25.72)	37 (20.33)	62 (43.66)
Admission method, *n* (%)											
Outpatient	22,576 (76.63)	114 (40.28)	2,843 (63.77)	4,698 (76.56)	4,809 (82.15)	5,020 (78.57)	3,195 (81.09)	1,501 (90.37)	222 (53.37)	146 (80.22)	28 (19.72)
Emergency	6,885 (23.37)	169 (59.72)	1,615 (36.23)	1,438 (23.44)	1,045 (17.85)	1,369 (21.43)	745 (18.91)	160 (9.63)	194 (46.63)	36 (19.78)	114 (80.28)
LOS, M (P_25_,P_75_)	7.01 (4.79,11.13)	21.19 (12.85,29.21)	8.95 (5.97,14.02)	5.92 (4.02,8.63)	6.10 (4.03,10.86)	7.00 (4.96,11.78)	8.09 (5.10,12.11)	7.03 (5.76,9.10)	8.06 (5.00,13.95)	7.15 (4.09,14.82)	13.73 (9.56,19.97)
Surgery, M(P_25_,P_75_)	1 (0,3)	5.(2,7)	0 (0,2)	2 (1,3)	2 (1,2)	1 (1,2)	2 (1,4)	1 (0,3)	0 (0,1)	0 (0,1)	2 (1,4)
CMI, M (P_25_,P_75_)	0.90 (0.65,1.89)	8.07 (8.07,8.07)	1.09 (0.71,1.67)	0.98 (0.81,2.89)	0.67 (0.53,1.10)	0.90 (0.82,1.68)	0.95 (0.59,2.48)	0.90 (0.59,1.83)	0.57 (0.46,1.42)	0.71 (0.56,1.36)	3.88 (2.50,5.97)

MDC, major diagnostic category; O/E, observed-to-expected; LOS, length of stay; CMI, case mix index.

### 3.2 Description of the number of CT scans in the top 10 MDCs

As shown in Table 2, MDCA and MDCZ had higher number of CT scans, with mean values of 2.58 and 2.92, respectively, and a median of 2 for both. For the remaining MDCs, the mean number of CT scans ranged from 0.96 to 1.84, with medians of either 0 or 1.

**Table 2:** Basic information about the number of CT scans in the top 10 MDCs.

MDC	Number	*x̅* ± s	M (P_25_,P_75_)	Max	Min
MDCA	729	2.58 ± 2.92	2 (1.4)	25	0
MDCB	8,195	1.84 ± 2.33	1 (0,2)	21	0
MDCF	8,379	1.37 ± 0.96	2 (1,2)	10	0
MDCG	5,588	0.96 ± 1.03	1 (0,1)	15	0
MDCH	6,456	1.01 ± 1.11	1 (0,2)	21	0
MDCI	5,325	1.35 ± 1.22	1 (0,2)	12	0
MDCK	1,885	1.13 ± 0.98	1 (1,1)	8	0
MDCS	495	1.19 ± 1.42	1 (0,2)	10	0
MDCT	206	1.13 ± 0.99	1 (0,2)	6	0
MDCZ	414	2.92 ± 2.97	2 (1,4)	15	0
Total	37,672	1.28 ± 1.42	1 (0,2)	25	0

### 3.3 Selection of risk-adjusted models for the number of CT scans

The overdispersion test statistic O was 69.83 and *P* value was less than 0.001, indicating significant overdispersion in the number of CT scans. The Vuong test statistic Z was 13.94 (*P* < 0.001), suggesting the presence of excess zero counts.

As shown in Table 3, the Poisson regression model demonstrated the poorest data fit. A comparison of the goodness-of-fit metrics across the four regression models revealed that the Zero-Inflated Negative Binomial (ZINB) model had the highest log-likelihood value and the lowest Akaike Information Criterion (AIC) and Bayesian Information Criterion (BIC) values. Therefore, the ZINB model was determined to be the optimum model for fitting the number of CT scans.

**Table 3:** The fitting goodness statistics of regression models of CT scans.

Models	AIC	BIC	Log likelihood
Poisson	83,523.50	83,639.57	−41,747.75
Negative binomial	83,062.17	83,186.54	−41,516.09
Zero-inflated Poisson	82,549.42	82,781.57	−41,246.71
Zero-inflated negative binomial	82,285.67	82,526.11	−41,113.84

### 3.4 Analysis of factors affecting the number of CT scans

The regression coefficients of the ZINB model are presented in Table 4. Within the ZINB model, the influence of risk factors on the number of CT scans can be evaluated from two perspectives: whether a CT scan was performed and the extent of CT scanning. The logit section on the left side of the table pertains specifically to the probability of zero counts. It was evident that gender, age, admission condition, first-time hospitalization status, presence of complications or comorbidities, and LOS were the risk factors of whether a patient underwent CT scanning. Male patients, older age, more stable admission condition, first-time hospitalization, higher comorbidity burden, and longer LOS were associated with an increased likelihood of receiving a CT scan.

**Table 4:** Risk adjustment—zero inflation negative binomial regression coefficient for CT scans.

Covariates	Logit section			Negative binomial section		
	OR (95%CI)	*Z*	*P*	IRR^a^ (95%CI)	Z	*P*
Gender						
Female (ref.)						
Male	0.533 (0.412,0.691)	4.766	<0.001	0.983 (0.961,1.005)	1.525	0.127
Age	0.939 (0.931,0.948)	13.730	<0.001	1.005 (1.004,1.006)	11.383	<0.001
Admission condition						
Dangerous (ref.)						
Urgent	0.083 (0.044,0.157)	7.663	<0.001	0.682 (0.639,0.728)	−11.577	<0.001
Stable	0.140 (0.070,0.279)	5.604	<0.001	0.667 (0.622,0.715)	11.484	<0.001
First-time hospitalization						
No (ref.)						
Yes	0.493 (0.378,0.643)	5.222	<0.001	1.149 (1.120,1.178)	10.604	<0.001
Critical and serious condition						
No (ref.)						
Yes	1.506 (0.944,2.404)	−1.717	0.086	1.173 (1.139,1.209)	9.673	<0.001
Medical insurance(%)						
Yes (ref.)						
No	1.983 (1.442,2.728)	4.210	<0.001	1.124 (1.077,1.172)	0.968	<0.001
Complication or comorbidity						
None (ref.)						
Mild	0.291 (0.199,0.425)	6.381	<0.001	1.013 (0.986,1.041)	3.624	0.333
Severe	0.322 (0.200,0.516)	4.698	<0.001	1.058 (1.026,1.092)	0.908	<0.001
Admission method						
Outpatient (ref.)						
Emergency	3.096 (2.045,4.687)	5.342	<0.001	0.983 (0.948,1.020)	16.574	0.364
LOS	0.516 (0.468,0.570)	13.234	<0.001	1.027 (1.025,1.028)	35.282	<0.001
Surgery	1.052 (0.958,1.158)	1.057	0.276	1.024 (1.019,1.029)	9.673	<0.001
CMI	1.026 (0.896,1.174)	0.365	0.715	1.049 (1.043,1.054)	16.574	<0.001

a Incidence Rate Ratio.

The negative binomial section on the right side of the table indicated that age, first-time hospitalization status, critical and severe illness status, medical insurance, comorbidity level, LOS, number of surgical procedures, and CMI were all influential factors affecting the number of CT scans. Several factors were associated with an increase in the number of CT scans, including advanced age, first-time hospitalization status, presence of critical or severe illness, absence of medical insurance, higher comorbidity burden, prolonged LOS, increased number of surgeries, and a higher CMI.

### 3.5 Evaluation of the rationality of the number of CT scans after risk adjustment

**Figure 2:**
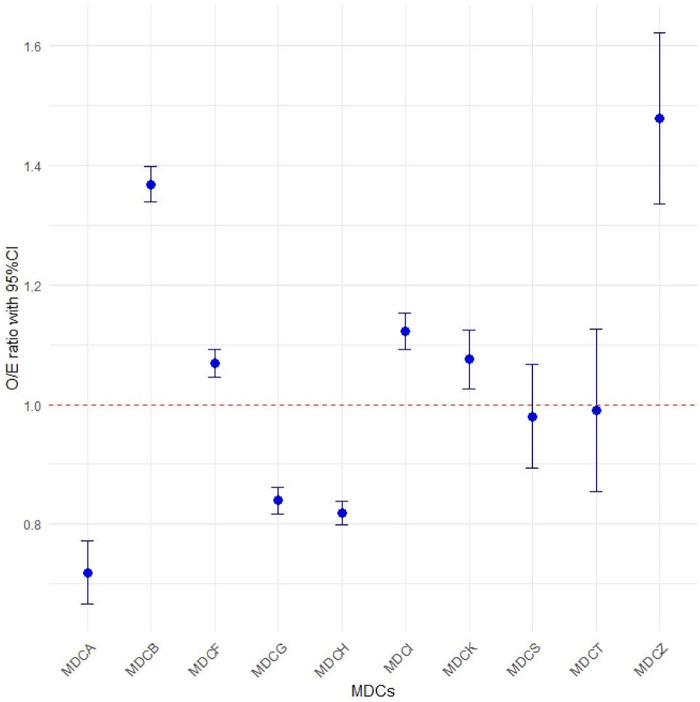
Observed-to-Expected (O/E) ratios for the number of CT scans in the top 10 MDCs.

As shown in Figure 2, the observed number of CT scans significantly differed from the risk-adjusted expected values across eight MDCs. The risk-adjusted O/E ratios ranged from 0.719 in the MDCA to 1.480 in the MDCZ. The O/E ratios for the MDCB, MDCF, MDCI, MDCK, and MDCZ were significantly greater than 1, indicating over-scanning of CT scans. In contrast, MDCA, MDCG, and MDCH had O/E ratios significantly less than 1, indicating under-scanning. The O/E ratios for the MDCS and MDCT were close to 1, reflecting rational CT scanning.

## 4 Discussion

This study utilized the ZINB model as a risk-adjusted model to evaluate the rationality of the number of CT scans. The ZINB model can effectively deal with the issues of overdispersion and excess zero counts commonly present in healthcare utilization data (26, 27), thereby meeting the statistical requirements of the number of CT scans characteristics in this study and enabling more scientifically valid comparisons of CT scan rates across different MDCs. The study focused specifically on the top 10 MDCs with the highest number of CT scans to enhance the relevance of the study.

We discussed the influencing factors of CT scans and explored the reasons affecting the number of CT scans. García-Albéniz et al. (23) reported that among Medicare patients in the United States, 7% underwent repeated abdominal or pelvic CT scans during the staging process of localized colorectal cancer. This indicates that Medicare patients receiving repeat scans were weaker and more complex, which may warrant the additional scans. In our research that doctors order more tests for insured patients to increase their income, leading to an increase in the number of CT scans. Our finding is consistent with this result. Higher out-of-pocket costs significantly decrease the likelihood of patients undergoing imaging examinations, especially in non-emergency situations. For example, a study on mammography screening demonstrated that any out-of-pocket expenses significantly reduced the likelihood of subsequent screening (30). Bellolio et al. (24) reported that the use of CT scans in emergency departments nearly doubled over the past decade, particularly among male patients, the elderly, and those patients presenting with pain, neurological symptoms, or trauma. The longer LOS, the higher the complexity and uncertainty of the patient's condition may be. In such cases, Doctors may be more inclined to schedule multiple CT scans to closely monitor patients' conditions, assess treatment effectiveness, and exclude potential complications. However, as noted by Ha et al. (25). The increase in CT scans may be associated with the increasing complexity of hospitalized patients' conditions. While CT scans may help shorten LOS, they may also be associated with higher readmission rates.

We incorporated CMI into the risk-adjustment model. While higher CMI values are associated with an increased likelihood of undergoing CT scans, they do not reflect the medical necessity of such scans. CMI reflects the clinical complexity of a hospital's patient population and serves as an indicator of the relative costs and resources required to treat a given patient population (31). A higher CMI value indicates a higher level of difficulty in providing medical services (32), which also means that cases with higher CMI values typically require more imaging assessments. Agarwal et al. (33) analyzed trends in inpatient CT utilization at academic medical centers from 2002 to 2007 and found that the CMI-adjusted CT scan rate increased by 28.0% during this period. This increase suggested that patients with higher CMI values did indeed require more CT scans, which was related to the rise in case complexity and resource demands.

Under the CHS-DRG payment system, the rationality of the number of CT scans was systematically analyzed across different MDCs. The results identified significant over-scanning in categories such as MDCB, MDCF, MDCI, MDCK, and MDCZ. This over-scanning may be attributed to the following factors: The MDCB includes acute and critical conditions such as stroke and epilepsy, which often require repeated CT scans for close monitoring of disease progression, thereby increasing the total number of scans (34). Than et al. (35) pointed out that life-threatening conditions such as aortic dissection and pulmonary embolism in MDCF can cause death within minutes to hours. Early symptoms such as chest tightness, shortness of breath, and fatigue often overlap with those of benign diseases, prompting clinicians to order additional imaging to avoid missing potentially dangerous conditions. Obese patients in the MDCK often undergo CT scans due to s suspected fatty liver disease and gallbladder disease. Ma et al. (36) noted that abdominal CT is effective in visualizing pancreatic inflammation and surrounding tissue edema, making it a suitable imaging modality for patients with acute pancreatitis. However, this examination is overused in obese patients, resulting in unnecessary radiation exposure. While polytrauma patients in MDCZ often involve injuries to multiple body regions, many trauma centers use whole-body CT scans as a standard procedure for all polytrauma patients, even if the patient has mild clinical symptoms or clearly defined injuries (37), leading to over-scanning. For MDCs with excessive imaging, specialized reviews of CT requests within these groups can be organized by experts. This process evaluates whether indications align with clinical pathways and identifies potential alternative diagnostic methods. Such reviews provide clear objectives for optimizing clinical pathways and continuously improving rational imaging practices.

Insufficient CT scanning was observed in MDCA, MDCG, and MDCH. In the MDCA, post-tracheostomy patients often present with critical conditions and multiple complications risk (38), which makes CT scans technically challenging and therefore may be clinically reduced. In MDCG, diagnosing acute abdominal conditions, occult gastrointestinal bleeding, and early-stage intestinal ischemia remains challenging due to atypical symptom presentation. These conditions are often misdiagnosed as functional gastrointestinal disorders or urinary calculi, leading to delayed or omitted CT examinations (39). Within the MDCH, Küstner et al. (40) found that some mild biliary colic cases presenting solely with dyspepsia were misdiagnosed as functional gastrointestinal disorders. Subsequent under-scanning of CT scans may lead to severe complications like suppurative cholangitis or gallbladder perforation. It is worth noting that the number of CT scans in the MDCS and the MDCT was closest to the expected value. This may indicate that these MDCs face insufficient medical resources or inefficient diagnostic workflows. It is advisable to evaluate whether the MDCs requires additional equipment, streamlined appointment scheduling processes, or enhanced training in relevant imaging diagnostics. This approach prevents resource constraints from compromising healthcare quality and safeguards equitable access to medical care for patients.

The limitations of this study include constraints imposed by inherent limitations in the data and modeling methods, including potential biases and unmeasured confounders. LOS is not an optimal variable, and incorporating it into the model carries the risk of reverse causality. However, due to the lack of more direct severity scores in the medical record summary data, we hope to obtain more comprehensive variables such as detailed clinical diagnoses and treatments in future studies. Furthermore, the single-center design may limit the generalizability of the findings. We plan to address this limitation by conducting a multicenter study to validate the broad applicability of our conclusions.

## 5 Conclusions

In the context of CHS-DRG payment reform, this study selected CT scans as a representative example of large medical equipment to develop a risk-adjustment framework for evaluating whether the number of CT scans among inpatients in hospitals is rational, and provide methodological support for the evaluation of rational examinations for other medical examination in hospitals.

## Data Availability

The raw data supporting the conclusions of this article will be made available by the authors, without undue reservation.
